# Pharmacotherapy weight-loss interventions to prevent type 2 diabetes in overweight or obese adults and older adults: A protocol for systematic review and network meta-analysis

**DOI:** 10.1097/MD.0000000000024812

**Published:** 2021-03-19

**Authors:** Wenxiao Yang, Junru Wen, Fangfang Wu, Hai Zeng, Bing Guo, Li Ge

**Affiliations:** a Department of Science and Technology, Shanghai University of Medicine and Health Sciences, Shanghai; b The Second Clinical College, Guangzhou University of Chinese Medicine, Guangzhou; c School of Nursing and Health Management, Shanghai University of Medicine and Health Sciences, Shanghai; d College of Traditional Chinese Medicine, Jinan University, Guangzhou; e Shanghai University of Traditional Chinese Medicine, Shanghai; f Xinhua Hospital Affiliated to Shanghai Jiaotong University School of Medicine, Shanghai, China.

**Keywords:** network meta-analysis, obesity, protocol, systematic review, type 2 diabetes, weight-loss medications

## Abstract

**Background:**

Obesity is a worldwide problem and is associated with multiple negative health effects. Obesity also has a direct relationship with risk of diabetes. Several pharmacotherapy weight-reducing interventions have been employed to prevent type 2 diabetes (T2D) in overweight or obese adults and older adults. However, data with respect to comparative effectiveness are limited. To address this gap, in this study, evidence on benefits of anti-obesity agents for preventing diabetes will be systematically reviewed using a network meta-analysis.

**Methods:**

We will perform an online systematic search for randomized controlled trials (RCTs) investigating 5 FDA-approved anti-obesity agents for preventing T2D in obese or overweight adults and older adults through electronic databases of PubMed, Embase, and the Cochrane Library from inception until December 31, 2020. Two independent reviewers will screen titles, abstracts, and full-texts of all potentially eligible trials. Two authors working independently will abstract data on trial-, participant- and intervention-related characteristics. The primary outcome will be incidence of T2D. Secondary outcomes will be achievement of normoglycaemia in patients with prediabetes, percentage of individuals achieving at least 5% or 10% weight loss of their baseline weight. We will conduct pairwise meta-analyses for all outcomes included in this study. To determine comparative efficacy of multiple interventions, network meta-analysis with a frequentist random-effects model will be performed. Moreover, subgroup analyses and sensitivity analyses will be performed to assess the robustness of our findings. To evaluate publication bias, the comparison-adjusted funnel plot will be utilized. Stata version 14.0 and RevMan version 5.3.3 will be used to perform all statistical analyses.

**Results:**

This study will evaluate the effectiveness of weight-loss medications on T2D prevention in overweight or obese people.

**Conclusions:**

This study will generate meaningful findings for overweight or obese adults and older adults, clinicians, and policy-makers concerning the optimal anti-obesity pharmacotherapy to decrease risk of T2D.

**Study registration number:**

INPLASY202110104.

## 1 Introduction

Obesity is a worldwide epidemic related to adverse health consequences, imposing a huge burden on individual and public health.^[1]^ The prevalence of obesity has been increasing over the past 3 decades; approximately 921 million individuals had obesity globally in 1980, which increased to 2.1 billion in 2013.^[2]^ Obesity is heritable and predisposes to various co-morbidities, including type 2 diabetes (T2D), cardiovascular and cerebrovascular disease, kidney disease, cancers, polyneuropathy, and musculoskeletal disorders.^[1,3–9]^ Additionally, obesity is also considered to increase risk of death. In 2017, 4.72 million deaths and 148 million disability-adjusted life-years (DALYs) were attributed to overweight and obesity globally.^[10]^ Moreover, economical burden of this disease is enormous, costs from obesity are estimated as high as £27 billion a year in the UK.^[11]^ Therefore, for overweight and obesity, identifying efficacious long-term interventions is of paramount importance.^[12]^

There is strong and consistent evidence that effective obesity management produces clinically meaningful health benefits, especially for prevention and treatment of T2D, which is a chronic and growing health problem worldwide.^[13–15]^ Given the limitations of lifestyle modifications alone and bariatric surgery to achieve or maintain clinically meaningful weight loss, weight-reducing medications as adjuncts in obesity therapy are vital.^[12,16–18]^ Anti-obesity drugs based on behavior interventions were associated with effecting meaningful weight change and reducing risk of developing diabetes.^[12,19,20]^ Five anti-obesity drugs have been approved by the U.S. Food and Drug Administration (FDA) for long-term management in overweight or obese people with the presence of 1 or more obesity-associated co-morbidities (e.g., T2D, hypertension, and hyperlipidemia).^[12,15,20,21]^ The 5 FDA-approved weight-loss drugs include phentermine-topiramate, naltrexone-bupropion, orlistat, lorcaserin, and liraglutide. Most of them have been employed in recent randomized placebo-controlled trials (RCTs) of diabetes prevention programs.^[22–25]^ Nevertheless, there is a paucity of RCT evidence comparing active weight-loss drugs with each other, while it would be important for clinical decision-making. Data with respect to overall and comparative efficacy of these medications on diabetes prevention can inform policymakers, health care practitioners, and patients concerning the optimal strategy to reduce risk of T2D.

To address this knowledge gap, we plan to systematically review the literature for 5 FDA-approved anti-obesity agents for preventing T2D in overweight or obese people. In the systematic review, relative efficacy and adverse effects of each drug regarding T2D prevention will be compared utilizing the approach of network meta-analysis. To date, no network meta-analysis comparing weight-reducing drugs for T2D prevention has been conducted.

## 2 Methods

### 2.1 Study design and registration

We will follow the statements on the Preferred Reporting Items for Systematic Reviews and Meta-Analyses (PRSIMA) for RCTs and its extension guidelines for network meta-analysis.^[26,27]^ A priori-established protocol has been registered (registration number: INPLASY202110104). Reporting of our systematic review protocol has been organized according to PRSIMA statements for protocols (PRIMSA-P).^[28,29]^

### 2.2 Eligibility criteria

#### 2.2.1 Patients

Eligible patients will be adults (aged 18–64 years) or older adults (aged ≥65 years) who are obese (defined as body mass index [BMI] ≥30 kg/m^2^) or overweight (BMI between 25 and 29.9 kg/m^2^). Patients diagnosed with type 1 or type 2 diabetes will be excluded.

#### 2.2.2 Interventions and comparators

Comparisons of 5 FDA-approved weight-loss drugs with either placebo, no intervention or each other will be included. Consequently, eligible comparisons are as follows: weight-loss drug versus another weight-loss drug, placebo versus weight-loss drug, and no intervention vs weight-loss drug.

#### 2.2.3 Outcomes

The primary outcome will be incidence of T2D in patients. Secondary outcomes will be achievement of normoglycaemia in patients with prediabetes, weight loss outcomes, including percentage of individuals achieving at least 5% or 10% weight loss of their baseline weight.^[14,20,30]^ Safety outcome will be all-cause death.

#### 2.2.4 Type of studies

Only RCTs studying any of the 5 FDA-approved anti-obesity agents compared with either placebo, no intervention, or each other will be included in our study. In order to be included, studies have to report the incidence of T2D or achievement of reversion from prediabetes to normoglycaemia as a primary or secondary outcome. We will include studies with a minimum intervention duration of 12 weeks.^[31,32]^

### 2.3 Search strategy

An online systematic search will be conducted for eligible RCTs studying 5 anti-obesity agents for prevention of T2D among obese or overweight adults and older adults using electronic databases of PubMed, Embase, and the Cochrane Library. The search will be conducted from database inception until December 31, 2020. All searches will be restricted to human trials published in English. The terms which will be searched alone or in combination are as follows: obesity, obese, overweight, diabetes, T2D, dysglycaemia, hyperglycaemia, phentermine-topiramate, naltrexone-bupropion, orlistat, lorcaserin, liraglutide, risk, conversion, delay, and prevent, controlled clinical trial, and randomized controlled trial. The preliminary search strategies of PubMed database are presented in the supplement. The detailed search strategies will be developed in consultation with an academic librarian. In addition to these database searches, the references from relevant reviews, conference proceedings, and meta-analyses will be scrutinized to identify other potentially-relevant studies for inclusion.

### 2.4 Selection of studies

Two independent authors will review titles and abstracts of all potentially eligible trials in accordance with a priori inclusion criteria. After discarding clearly nonrelevant articles, the remaining studies identified will undergo further examination for full-text articles. Discrepancies in study selection procedures will be resolved by discussion. The details of study identification will be presented in the PRISMA flow chart.

### 2.5 Data collection process

Two authors independently will abstract data on trial-, participant- and intervention-related characteristics onto a standardized form. Disagreements will be resolved by consensus. The correspondence authors will be contacted to acquire unpublished data by e-mails, if necessary. Only trials with extractable data will be included, when no additional information could be obtained from relevant authors. Extracted data elements will include:

Baseline demographic characteristics (age, gender, race, weight, and BMI).
Trial specific data (author, publication year, study design, country setting, duration of treatment and follow-up, and financial support).
Details of interventions and comparators (administration route, dose, dosage, and frequency).
Numerical data of the primary outcome and secondary outcomes of interest.

### 2.6 Assessment of methodological quality

The risk of bias of eligible RCTs will be examined through the Cochrane Collaboration's tool by 2 authors. This instrument involves evaluation of the following domains: random sequence generation, allocation concealment, blinding of participants and personnel, blinding of outcome assessment, incomplete outcome data, selective reporting, and other sources of bias.^[33]^ Response options for each item will be low, unclear, or high risk of bias. Disagreements in quality assessment will be settled by consensus with a third reviewer.

### 2.7 Statistical analysis

#### 2.7.1 Pairwise meta-analysis

We will conduct standard pairwise meta-analyses for all outcomes involved in the review. Dichotomous variables will be expressed as risk ratios (RRs) with 95% confidence intervals (CIs). Continuous outcomes will be expressed as mean differences (MDs) and 95% CIs. The random-effects model will be applied to combine the results. A *P* value of less than .05 will be determined to be statistically significant. We will estimate statistical heterogeneity within each direct pairwise comparison through the *I*^*2*^ statistic. An *I*^*2*^ value of more than 75% will be set as substantial statistical heterogeneity.^[34,35]^ In the case of substantial statistical heterogeneity, we plan to present a narrative or tabulated summary instead of combining study results. Stata version 14.0 and RevMan version 5.3.3 will be used to conduct all statistical analyses.

#### 2.7.2 Network meta-analysis

We will perform network meta-analysis with the methodology of a frequentist random-effects model to determine comparative efficacy of multiple treatments. This approach permits evidence from direct and indirect comparisons. In the absence of direct head-to-head comparisons of interventions (e.g., B vs C), an indirect treatment comparison could be derived from studies comparing A vs B and A vs C. The “mvmeta” and “network” suite of commands in Stata software will be used for this analysis. The node-splitting approach will be used to detect the assumption of consistency locally.^[36,37]^ To evaluate the presence of inconsistency in the whole network, a “design-by-treatment” model will be applied.^[36]^ Moreover, we plan to rank medications in order of their efficacy according to the surface under the cumulative ranking (SUCRA)_._^[38]^ A higher SUCRA value represents better efficacy.

#### 2.7.3 Publication bias

We will construct the comparison-adjusted funnel plot to investigate reporting bias at the network level.^[39]^

#### 2.7.4 Sensitivity analyses

We plan to conduct sensitivity analyses regarding the primary outcome via excluding studies with overall unclear or high risk of bias.

#### 2.7.5 Subgroup analyses

Additional subgroup analyses will also be performed. Where the data permit, analyses will be stratified according to age (18–64 years and ≥65 years), sex, BMI (25–29.9 kg/m^2^ and ≥30 kg/m^2^), and sample size of trials (≥100 participants per arm and <100 participants per arm).^[40,41]^ Moreover, subgroup analyses regarding patients with normoglycaemia and prediabetes at baseline will also be performed.

### 2.8 Quality of evidence

The quality of evidence will be apprised with the grading of recommendations assessment, development and evaluation (GRADE) criteria.^[42]^ The evidence grade of each outcome will be classified as high, moderate, low, or very low. Several domains will lead to downgrading of the quality of evidence, including inconsistency, imprecision, indirectness, publication bias, and study limitations.

### 2.9 Ethics and dissemination

Formal ethical approval is not required given the absence of no individual patient data in this study. We plan to disseminate the findings through peer-reviewed publications.

## 3 Discussion

This study is the first comprehensive systematic review and network meta-analysis combining direct and indirect evidence from RCTs of weight-reducing drugs for T2D prevention. Currently, the FDA has approved 5s weight-loss medications for long-term management in overweight or obese adults and older adults with no less than 1 obesity-related complications. Whether anti-obesity agents can be prescribed for diabetes prevention among those patients is still controversial. This study will provide beneficial findings for overweight or obese adults and older adults, clinicians, and policy-makers with respect to the optimal anti-obesity agent to reduce risk of developing T2D. However, this review has several potential limitations. First, it is possible that data are derived predominantly from placebo-controlled trials with limited studies comparing 2 active weight-reducing agents against each other. The comparative efficacy of 5 FDA-approved weight-loss medications may be mainly estimated from indirect comparisons rather than direct comparisons, and the consistency between direct evidence and indirect evidence can be difficult to evaluate_._^[43–45]^ Second, the possibility of heterogeneity across studies may not be excluded, though predefined strict inclusion criteria are utilized to enhance comparability. Finally, only currently FDA-approved medications for long-term management in overweight or obese adults and older adults are analyzed in this study, other non-approved weight loss therapies are excluded.

## Author contributions

**Conceptualization:** Wenxiao Yang, Hai Zeng.

**Data curation:** Wenxiao Yang, Junru Wen, Li Ge.

**Methodology:** Wenxiao Yang, Hai Zeng.

**Software:** Wenxiao Yang, Junru Wen, Fangfang Wu, Hai Zeng.

**Writing – original draft:** Wenxiao Yang, Fangfang Wu, Hai Zeng, Bing Guo.

**Writing – review & editing:** Junru Wen, Bing Guo, Li Ge.

## Supplementary Material

Supplemental Digital Content
