# Supplementary material for: Pharmacotherapy weight-loss interventions to prevent type 2 diabetes in overweight or obese adults and older adults: A protocol for systematic review and network meta-analysis
Source: Medicine (Baltimore). 2021 Mar 19;100(11):e24812. doi: 10.1097/MD.0000000000024812 (PMC7982236; doi:10.1097/MD.0000000000024812)
Supplement: Supplemental Digital Content [file medi-100-e24812-s001.pdf]

Search strategy in PubMed.

Block 1: Obesity

#1 “obesity”[Mesh] OR “Anti-Obesity Agents”[Mesh] OR “weight loss”[Mesh] OR “overweight”[Mesh]

#2 obes*[tiab] OR “body mass ind*”[tiab] OR adipos*[tiab] OR overweight[tiab] OR “over weight”[tiab] OR “overload syndrom*”[tiab] OR overeat*[tiab] OR “over eat*”[tiab] OR overfeed*[tiab] OR “over feed*”[tiab] OR overfed[tiab] OR “over fed”[tiab] OR “weight cycling”[tiab] OR ((weight[tiab] OR fat[tiab]) AND (gain*[tiab] OR reduc*[tiab] OR los*[tiab] OR maint*[tiab] OR decreas*[tiab] OR watch*[tiab] OR control*[tiab])) OR “skinfold thickness”[tiab] OR antiobesity[tiab] OR “anti-obesity”[tiab] OR obesitas[tiab] OR bodyweight[tiab] OR “body weight”[tiab]

#3 #1 OR #2

Block 2: Prevention of T2DM

#4 “Diabetes Mellitus, Type 2”[Mesh] OR “diabetes mellitus type 2”[tiab] OR “type 2 diabetes mellitus”[tiab] OR “T2D*”[tiab] OR diabet*[tiab] OR NIDDM[tiab] OR “type 2”[tiab] OR “type II”[tiab] OR “dysglycaemia”[tiab] OR “hyperglycaemia”[tiab]

#5 risk[tiab] OR progress*[tiab] OR prevent*[tiab] OR inciden*[tiab] OR conversion[tiab] OR develop*[tiab] OR delay*[tiab]

#6 #4 AND #5

Block 3: Orlistat

#7 alli[tiab] OR orlipastat[tiab] OR orlistat[tiab] OR “ro 18 0647”[tiab] OR “ro 180647”[tiab] OR ro180647[tiab] OR tetrahydrolipstatin[tiab] OR xenical[tiab]

Block 4: Lorcaserin

#8 lorcaserin[tiab] OR “apd 356”[tiab] OR apd356[tiab] OR belviq[tiab] OR lorqess[tiab]

Block 5: Liraglutide

#9 liraglutide[tiab] OR “nn 2211”[tiab] OR nn2211[tiab] OR “nnc 90 1170”[tiab] OR “nnc90 1170”[tiab] OR Saxenda[tiab] OR victoza[tiab]

Block 6: Phentermine-topiramate

#10 (phentermine[tiab] AND topiramate[tiab]) OR phenterminetopiramate[tiab] OR qnexa[tiab] OR qsiva[tiab] OR qsymia[tiab] OR topiramatephentermine[tiab]

Block 7: Naltrexone-bupropion

#11 (amfebutamone[tiab] AND naltrexone[tiab]) OR (bupropion[tiab] AND naltrexone[tiab]) OR contrave[tiab]

Block 8: RCT-filter

#12 “randomized controlled trial”[pt] OR “controlled clinical trial”[pt] OR randomized[tiab] OR placebo[tiab] OR “drug therapy”[sh] OR randomly[tiab] OR trial[tiab] OR groups[tiab]

Block 9:

#13 #3 AND #6 AND (#7 OR #8 OR #9 OR #10 OR #11) AND #12
